# Screening *PAX9*, *MSX1* and *WNT10A* Mutations in 4 Iranian Families with Non-Syndromic Tooth Agenesis

**Published:** 2020

**Authors:** Shiva Safari, Asghar Ebadifar, Hossien Najmabadi, Koorosh Kamali, Seyedeh Sedigheh Abedini

**Affiliations:** 1. Tehran, Iran; 2. Dentofacial Deformities Research Center, Research Institute of Dental Sciences, Department of Orthodontic, Faculty of Dentistry, Shahid Behehsti University of Medical Sciences, Tehran, Iran; 3. Genetics Research Center, University of Social Welfare and Rehabilitation Sciences, Tehran, Iran; 4. Department of Public Health, Faculty of Public Health, Zanjan University of Medical Sciences, Zanjan, Iran

**Keywords:** Iran, *MSX1*, Mutation, *PAX9*, Tooth agenesis, *WNT10A*

## Abstract

**Background:**

Tooth agenesis is one of the most common developmental anomalies in human and the main reasons for its occurrence are still unknown. Mutations of several genes such as *PAX9*, *MSX1*, *AXIN2*, *KDF1* and *WNT10A* have been reported which are associated with non-syndromic tooth agenesis. However, *PAX9*, *MSX1* and *WNT10A* are commonly reported in the literature. Hence, the aim of this study was to investigate the mutations of these genes in 4 Iranian families with non-syndromic tooth agenesis.

**Methods:**

DNA extractions from peripheral blood cells of patients with non-syndromic tooth agenesis from 4 unrelated Iranian families were performed by salting out method, and the candidate genes were amplified then followed by Sanger sequencing method.

**Results:**

One missense variant (rs4904210) and 4 Single Nucleotide Polymorphisms (SNPs) (rs2236007, rs12883298, rs12882923 and rs12883049) were found in *PAX9* gene. Five variants (rs149370601, rs8670, rs186861426 and rs774949973) including a missense variant (rs36059701) were detected in *MSX1* gene and no variants were found in *WNT10A* gene.

**Conclusion:**

All variants were analyzed based on bioinformatics websites and Iranian gene databases, and as a result, it was revealed that variants of *PAX9*, *MSX1* and *WNT10A* may not play a role in non-syndromic tooth agenesis among Iranian cases.

## Introduction

Tooth agenesis is one of the most common developmental anomalies in human which can cause aesthetic, masticatory and functional problems ^1^. Third molar is the most affected tooth, followed by maxillary lateral incisor and mandibular second premolar ^2^.

Prevalence of congenitally missing teeth in Iranian population is estimated to be approximately 10.9%. The most frequent congenitally missing teeth are mandibular second premolars (23.34%) followed by maxillary second premolars (22.02%) ^3^.

Dental agenesis can occur in different ways. It can be a part of a syndrome such as Ectodermal dysplasia or happens as an isolated trait ^4^. Tooth agenesis classification in the literature is according to the number of missing teeth; hypodontia refers to the condition when there are one to five missing teeth and in oligodontia, six or more teeth are missing excluding third molars ^5^.

Among several genes involved in tooth development, mutations in *MSX1*, *PAX9*, *AXIN2*, *WNT10A*, *EDA* and *KDF1* have been reported to play a role in tooth agenesis ^6–13^. *MSX1* is the first gene detected to have an association with this anomaly ^6^. A heterozygous mutation in either *PAX9* or *MSX1* has been widely reported to cause tooth agenesis in populations ^6,11,13–16^.

*MSX1* belongs to Homeobox family and is the first gene reported in association with tooth agenesis ^17^. *PAX9* belongs to the family of paired box ^18^, and is the primary gene involved in early tooth development in humans ^13^. *MSX1* and *PAX9* are two transcription factors which are expressed in dental mesenchyme in response to epithelial signals and they play an important role during progression from bud to cap stage in odontogenesis ^6,19^. *WNT10A* mutations in ectodermal dysplasia were reported in autosomal dominant and recessive forms ^20^. The mutations of these genes can induce severe hypodontia and non-syndromic tooth agenesis ^8,20^. The only consistent feature of patients with *WNT-10A* mutations is hypodontia ^21^.

Lack of studies in this field despite the high prevalence in Iran necessitates studies to find the exact causative factors in the affected population. The aim of the present study was to identify the mutations in the candidate genes, *PAX9*, *MSX1* and *WNT10A*, responsible for tooth agenesis in 4 Iranian families with hereditary pattern of non-syndromic tooth agenesis.

## Materials and Methods

### Pedigree construction and clinical diagnosis

Members of 4 unrelated Iranian families with 2 or more individuals affected with tooth agenesis were selected through clinical and radiographic examinations. Informed consent forms were obtained from all participants and the study was approved by the Ethics committee of Shahid Beheshti University of Medical Sciences (IR.SBMU.DRC.REC.1398.085).

### DNA extraction and sequencing

Peripheral blood samples of all the participants were obtained and genomic DNA was extracted by salting out method. Forward and reverse primers were designed for all coding and flanking regions of *PAX9*, *MSX1* and *WNT10A* genes, and amplified by Polymerase Chain Reaction (PCR) (Tables 1–3).

**Table 1.** *MSX1* designed primers

**Name**	**Sequence**
MSX1-ex.1.a-F	CTTCAGCGCAGAGGAAAGTTTCC
MSX1-ex.1.a-R	AGGAGCGAAGGGGACACTTTG
MSX1-ex.1.b-F	CTCGGTGTCAAAGTGGAGGAC
MSX1-ex.1.b-R	CAAGGCGAGGAGGTCTGGAA
MSX1-ex.2-F	TCTTGGGCTGATCATGCTCC
MSX1-ex.2-R	GCCCTCAGTTTCCCCATCTT

**Table 2.** *PAX9* designed primers

**Name**	**Sequence**
PAX9-ex.2-F	ACATTCAGACCAAACGCTTTCA
PAX9-ex.2-R	TGTAGGAACACGAGCAAAGTCA
PAX9-ex.3.a-F	GATTGGACAGTGACGGTTTGG
PAX9-ex.3.a-R	CCGATCTTGTTGCGCAGAAT
PAX9-ex.3.b-F	AAGATCCTGGCGCGATACAA
PAX9-ex.3.b-R	TCAGGTGGTGGGAAAGACAG
PAX9-ex.4-F	GGTCAGAGAATTTGGAAAGGCCT
PAX9-ex.4-R	CTCGTAGCAGCAAAGGGACG
PAX9-ex.5-F	TGTTTAAGTGGCAAGACTGTGA
PAX9-ex.5-R	CCTTTGAGGGGTGTAGGTTTCT

**Table 3.** *WNT10A* designed primers

**Name**	**Sequence**
WNT10A-ex.1-F	CTTGAGAGGCACCGGGAGTT
WNT10A-ex.1-R	CTTGTGCTTACTCCTGAGGTGG
WNT10A-ex.2-F	GGAGGTCAAGGAGAGAGGAATGT
WNT10A-ex.2-R	GTGTGTGGGGATGGGAGGAT
WNT10A-ex.3-F	TGCCCTTCTTTGACTCTGTTTCC
WNT10A-ex.3-R	AACGAGAAGATTGCCAAGGTGT
WNT10A-ex.4-F	AGAAGTTCTTCTGACTGCCTGG
WNT10A-ex.4-R	AAGAACCCAGTCAGTCCTAGAG

PCR reaction mix was performed by forward and reverse primers with concentration of 10 *pmol* and 50–100 *ng* of template DNA in total volume of 20 *μmol* using Super PCR MasterMix 2X (Yektatajhiz, Iran). The PCR was started at 95*°C* for 5 *min* followed by 20 cycles (95*°C* for 40 *s*, 65*°C* for 30 *s* with a decrement of 0.5*°C* per cycle, 72*°C* for 40 *s*) and in the second step by 30 cycles (95*°C* for 40 s, 55*°C* for 30 *s* and 72*°C* for 40 *s*). The process was concluded by a 5 *min* extension at 72*°C.* Then, mutation screening for all subjects was performed using direct Sanger sequencing (ABI PRISMTM3100; Applied Biosystems, Foster City, CA, USA).

### Mutation analysis

Allele frequency of all variants was obtained using https://asia.ensembl.org/, https://genome.ucsc.edu/, https://www.internationalgenome.org/1000-genomes-browsers/ and http://www.iranome.ir/websites.

Protein prediction was checked *via* polyphen 2 and SIFT. Finally, the pathogenicity scores of variants were determined by the information available at https://www.ncbi.nlm.nih.gov/clinvar/, https://varsome.com/, http://wintervar.wglab.org/websites, augmented with the data available at http://www.iranome.ir/. All variants were interpreted according to ACMG guidelines.

## Results

### Clinical findings

**Figure 1.**
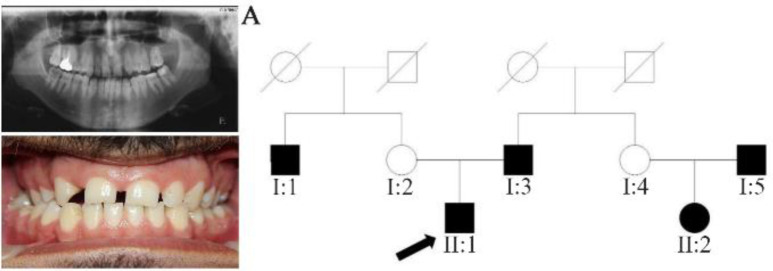
Pedigree of A family. Panoramic radiograph and the clinical photograph of the proband.

**Figure 2.**
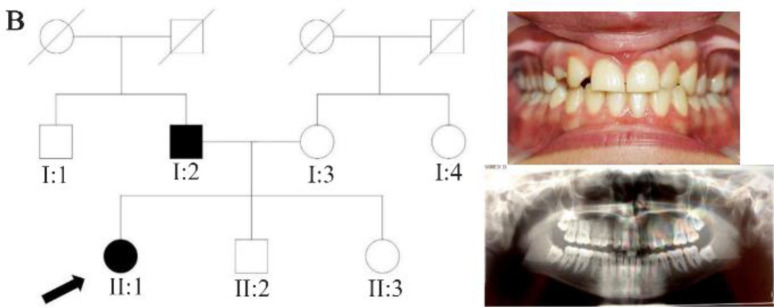
Pedigree of B family. Panoramic radiograph and the clinical photograph of the proband.

**Figure 3.**
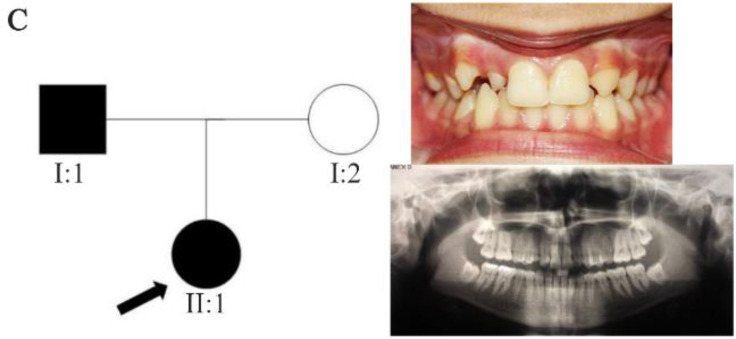
Pedigree of C family. Panoramic radiograph and the clinical photograph of the proband.

**Figure 4.**
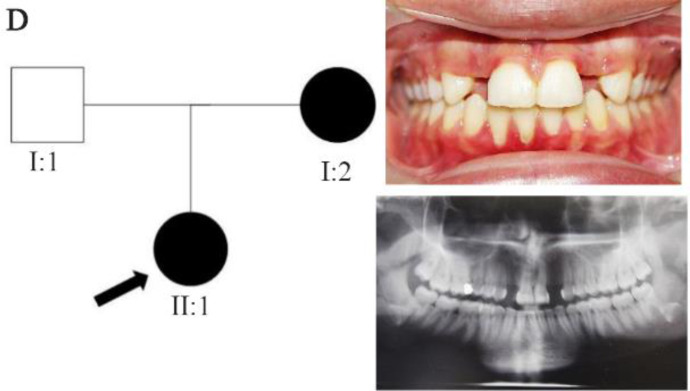
Pedigree of D family. Panoramic radiograph and the clinical photograph of the proband.

In family A, patient II: 1 had a missing maxillary lateral incisor, the same as the father (Patient #I: 2) (Figure 1). Patient II: 2 in the first family had second premolar agenesis, the same as his father (I: 5). The trait seems to be unrelated to the proband. Right lateral incisors of patient II: 1 and patient I: 2 of family B were missing (Figure 2). Both the proband of family C (II: 1) and her father (I: 2) had missing right maxillary lateral incisor (Figure 3). Patient II: 1 of family D had both missing maxillary lateral incisors, the same as mother (Patient #I: 2) (Figure 4). All the patients were examined for other craniofacial anomalies and syndromes.

### Sequencing analysis

In *PAX9* gene, three reported heterozygous variants were found in 5′ UTR(rs12883298 and rs12882923 in A, B and D and rs12883049 in A and D family) and one heterozygous intronic variant (rs2236007) was found in A family. A heterozygous missense variant in exon 4 (rs4904210) was detected in all affected members of A, B and D family.

*MSX1* sequencing revealed one heterozygous missense variant in exon 1a (rs36059701) in C family, one intronic variant in exon 1b (rs149370601) in C, A (heterozygous) and B family (Homozygous). Two heterozygous variants in 5′UTR in exon 1b (rs186861426 in A family and rs774949973 in C family) and one heterozygous 3′ UTR variant in exon 2(rs8670) in A and C family were also found. All the variants were reported to be Single Nucleotide Polymorphism (SNP) and classified as non-pathogenic. No variants in *WNT10A* gene were found in the families.

## Discussion

Tooth agenesis is mostly seen in non-syndromic pattern ^22^. Several genes, including *MSX1*, *PAX9*, *AXIN2*, *WNT10A* and *EDA* have been reported to involve in tooth agenesis. *PAX9* mutations tend to affect molars, whilst *MSX1* mutations cause second premolar agenesis ^18^. *EDA* mutations mainly affect anterior teeth ^9^.

A missense variant (rs4904210) was identified in this study. This variant was previously reported as a benign variant in *PAX9* gene ^6^. In a meta-analysis performed by Zhang *et al*, there was no association between this SNP and tooth agenesis ^14^.

Another study performed in 2018 by Zhang *et al* investigating novel mutations in *PAX9* gene suggested that the SNP (rs4904210) with the novel mutation (c.G1057A) can induce microdontia and hypodontia ^11^.

In a review accomplished by Bonczek *et al* on *PAX9* mutations and tooth agenesis, they suggested the possibility of an association between rs4904210 and non-syndromic hypodontia, at least in males ^23^.

Wang *et al* in screening of a family with 6 individuals represented no mutations in *MSX1* and *AXIN2* genes, leading to a conclusion that rs4904210 polymorphism could be a risk factor for tooth agenesis in Han ethnicity ^24^. In contrast, Pereira *et al* suggested there was no association between rs4904210 and tooth agenesis, and the variant is mostly a neutral polymorphism in humans ^25^.

*PAX9* mutations reported to affect molars and premolars ^15,18^ while mutations in *PAX9* can have an association with agenesis of different kinds of teeth ^26^. Since in our study the most prevalent missing tooth was lateral incisor and two families had different phenotypes specially patients I: 5 and II: 2 in the first family with premolar agenesis, the existence of variant rs4904210 in all of the patients may suggest that this variant cannot be the main cause for tooth agenesis; however, the possibility also cannot be definitely rejected.

Other studies reported the existence of 3 variants in 5′ UTR variant (rs12883298, rs12882923 and rs12883049) ^6^, with high allele frequency (0.552 for rs12883298 and 0.0552 for rs12882923 and 0.207 for rs12883049). The intronic variant (rs2236007) has high allele frequency of 0.189. All these variants have high frequency which indicates these variants are common in Iranian population. Although a study performed in Czech Republic and 2 other studies in Tunisian and Brazilian populations reported the polymorphisms of rs12883298 and rs12883049 may have possible influences in hypodontia occurrence, further researches with larger groups should be implemented to confirm the findings ^15,27,28^.

The missense variant (rs36059701) on *MSX1* gene has been reported to play a role on orofacial clefts ^29^ and also the high prevalence (0.1036) suggests that this variant cannot be responsible for tooth agenesis in our studied population.

The rs8670 variant in 3′ UTR has previously been reported to be associated with non-syndromic tooth agenesis ^30,31^. Another study suggested that this variant, in homozygous form, may contribute to agenesis of upper lateral incisors; however, since the polymorphism is quite common, additional genes must be involved in this phenotype ^32^.

## Conclusion

In conclusion, the variants found in the *PAX9*, *MSX1* and *WNT10A* genes may not play a role in nonsyndromic tooth agenesis, but further studies should be performed to explore the exact association between these SNPs and the disease. The other candidate genes also should be assessed in order to find the exact causative relation for tooth agenesis.
